# Composition of agarose substrate affects behavioral output of Drosophila larvae

**DOI:** 10.3389/fnbeh.2014.00011

**Published:** 2014-01-28

**Authors:** Anthi A. Apostolopoulou, Fabian Hersperger, Lorena Mazija, Annekathrin Widmann, Alexander Wüst, Andreas S. Thum

**Affiliations:** Department of Biology, University of KonstanzKonstanz, Germany

**Keywords:** *Drosophila* larva, agarose, gustation, bitter, choice behavior, feeding, learning and memory

## Abstract

In the last decade the *Drosophila* larva has evolved into a simple model organism offering the opportunity to integrate molecular genetics with systems neuroscience. This led to a detailed understanding of the neuronal networks for a number of sensory functions and behaviors including olfaction, vision, gustation and learning and memory. Typically, behavioral assays in use exploit simple Petri dish setups with either agarose or agar as a substrate. However, neither the quality nor the concentration of the substrate is generally standardized across these experiments and there is no data available on how larval behavior is affected by such different substrates. Here, we have investigated the effects of different agarose concentrations on several larval behaviors. We demonstrate that agarose concentration is an important parameter, which affects all behaviors tested: preference, feeding, learning and locomotion. Larvae can discriminate between different agarose concentrations, they feed differently on them, they can learn to associate an agarose concentration with an odor stimulus and change locomotion on a substrate of higher agarose concentration. Additionally, we have investigated the effect of agarose concentration on three quinine based behaviors: preference, feeding and learning. We show that in all cases examined the behavioral output changes in an agarose concentration-dependent manner. Our results suggest that comparisons between experiments performed on substrates differing in agarose concentration should be done with caution. It should be taken into consideration that the agarose concentration can affect the behavioral output and thereby the experimental outcomes *per se* potentially due to the initiation of an escape response or changes in foraging behavior on more rigid substrates.

## Introduction

*Drosophila* larvae are able to express a comprehensive set of sophisticated behaviors to perceive their environment as well as to orientate and locate in it (Gerber and Stocker, 2007; Gerber et al., 2009). Based on their simple neuronal architecture and genetic amenability, larvae are used as a model organism to identify the neuronal basis of these behaviors up to the single neuron level (Fishilevich et al., 2005; Selcho et al., 2009, 2012; Pauls et al., 2010b; Keene et al., 2011). Nearly all of these behavioral approaches in larvae have in common the use of Petri dishes filled with a layer of agar or agarose as a substrate for crawling and for preventing dehydration.

Agar is derived from agarophyte seaweeds, primarily from *Gelidium* and *Gracilaria* species (Food and Agriculture Organization of the United Nations; http://www.fao.org), where it accumulates in the cell walls. Agar is a mixture of at least two polysaccharides (Araki, 1937); agarose, which has a solidifying property, and agaropectin. In most behavioral studies, agarose is preferred for substrate preparation, as its high purity allows for a standardized mixture among different experiments and experimental trials (Fishilevich et al., 2005; Michels et al., 2005, 2011; Pauls et al., 2010a; Keene et al., 2011; Schleyer et al., 2011; Von Essen et al., 2011; El-Keredy et al., 2012; Huser et al., 2012; Apostolopoulou et al., 2013). However, in some cases agar is used, mainly because of its lower cost compared to pure agarose (Khurana et al., 2009, 2012).

To our knowledge, so far no study has parametrically investigated the role of agarose concentration on larval behavior. In detail, five randomly chosen larval behavioral studies used either 0.5% agar (Khurana et al., 2009, 2012), or agarose at concentrations of 1.4% (Aceves-Pina and Quinn, 1979), 1.0% (Luo et al., 2010), or 1–2.5% (Rohwedder et al., 2012). Thus, agarose concentration varied in different studies while using the same behavioral assay. In some cases, different agarose concentrations were applied within a single study (Rohwedder et al., 2012). And also, certain reports are based on different agar substrates (Khurana et al., 2009, 2012), affecting both the quality and the concentration of the substrate. Additionally, many studies lack details about the products used. In conclusion, the concentration and quality of the used agar/agarose substrate was often completely neglected. Thus, as long as the effects of these parameters on larval behavior are not thoroughly analyzed, results obtained in different studies using different agarose concentrations may not be comparable.

Here, we have used ultrapure agarose to investigate whether different agarose concentrations have an effect on larval behavior. To this end, we have applied assays based on sensation and processing of agarose alone as well as on sensation and processing of the bitter substance quinine mixed in an agarose solution. More specifically, we performed preference, feeding, learning and locomotion assays. To our surprise we found that the behavioral outputs of all four paradigms tested depend on the agarose concentration. Higher agarose concentrations increase larval crawling speed while reducing gustatory-driven behavioral output. Thus, on a rigid substrate that prevents animals from burrowing into the medium it is tempting to speculate that larvae express an escape behavior that represses choice behavior, feeding and learning.

## Materials and methods

### Fly stock and maintenance

Wild type CantonS (WTCS) flies were raised on standard *Drosophila* medium at 25°C. For all behavioral experiments, flies were transferred to new vials and allowed to lay eggs for two days. The experiments were performed 5 or 6 days after egg laying. Only feeding stage larvae were used, in groups of 25–30 animals or as individuals.

### Choice behavior

To prepare the agarose solution ultrapure agarose (UltraPure™ Agarose; Invitrogen; Catalog number 16500500) in ddH2O was heated up in a microwave. Agarose-quinine mixtures were prepared by adding 6 mM quinine (quinine hemisulfate; Sigma Aldrich; Q1250) in the hot agarose solution and stirring adequately. To make choice behavior plates, petri dishes were filled with agarose (and when applicable quinine) solution. After cooling down, the agarose (-quinine) solution was subsequently removed from the one half of the plate. This half was then refilled with a second agarose (-quinine) solution. The concentration of agarose [ranging from 0.5–3.5% (w/ml)] and the addition of quinine in the mixtures varied as described for each experiment in the respective part of the results. During the choice assay the larvae were placed in the middle of the plate along the vertical axis and were left to move freely for 5 min. After this time was up, the larvae on one side of the plate (side A), on the opposite side (side B) and in the middle were counted. As a middle zone we define a 1 cm zone in the middle of the plate where the larvae were placed at the beginning of the experiment. The Preference Index for each measurement was calculated as follows:

Preference Index=(#sideA−#sideB)/#total

Negative Preference Indices indicate avoidance behavior towards side A.

### Quinine diffusion

Petri dishes containing either agarose alone or agarose in different concentrations and 6 mM quinine were prepared (for details in the concentrations please refer to the results). Taking into account that quinine solutions are highly fluorescent at about 460 nm (for details see http://www.olympusmicro.com/primer/techniques/fluorescence/fluorescenceintro.html) the plates were analyzed under UV light in BioDocAnalyzer (Biometra) and photos were taken. The mean pixel intensity of two defined spots of the same pixel size (~700 pixel) per plate were defined as region of interest (ROI). Per single plate one spot was always located in the center of the plate and one in the periphery along the same longitudinal axis to guarantee a similar illumination. Mean values for each ROI were calculated using Fiji. In each case 10 plates were analyzed.

### Feeding

Petri dishes used for analyzing feeding behavior on pure agarose were filled with a solution of different agarose concentrations ranging from 0.5 to 3.5% (w/ml), and 2% (w/ml) indigo carmin (Sigma Aldrich cat. no.: 73436). Petri dishes used for analyzing feeding behavior on quinine containing substrates were filled with a solution of agarose at various concentrations, 2% (w/ml) indigo carmin and 6 mM quinine. For details on the used concentrations please refer to the respective part in the results. During the feeding assay larvae of all groups were allowed to feed on the substrate for 30 min, afterwards they were washed in tap water and homogenized in 500 μl of 1 M ascorbic acid solution (Sigma Aldrich cat. no.: A7506). The homogenate was centrifuged for 5 min at 13′400 rpm. The supernatant was filtered using a syringe filter (millipore, 5-μm pores) into a new Eppendorf cup and then centrifuged again for 5 min at 13′400 rpm. 100 μl of the supernatant was loaded on a 96-well plate (Hartenstein, Würzburg, Germany). The absorbance at 610 nm of each well mixture was measured using an Epoch spectrophotometer (BioTek, Bad Friedrichshall, Germany). The final absorbance of each single measurement was calculated by deducting the mean absorbance of the blank control (1 M ascorbic acid) from the absorbance of the relative mixture.

$$\begin{array}{l} (\text{Final})\text{Absorbance}=\text{absorbance of the mixture}\\ \;-\text{absorbance of the blank control} \end{array}$$

To calculate the normalized absorbance, the final absorbance of the larvae fed with 6 mM quinine in a specific agarose concentration was divided by the absorbance of the larvae fed with pure agarose solution in the same concentration.

$$\begin{array}{l} (\text{Final})\text{ Normalized absorbance}=(\text{Final}){\text{ absorbance}}_{\text{6 mM quinine}}\\ \;/(\text{Final}){\text{absorbance}}_{\text{pure}} \end{array}$$

### Tracking

Petri dishes containing agarose solutions in different concentrations ranging from 0.5 to 3.5% (w/ml) were prepared. Individual larvae were positioned in the center of the plate and their locomotion was recorded using a Basler GigE Vision Camera scA1300-32 gm (objective Fujinon TV Lens HF12.5 HA-1B 1:1.4/12.5 mm) set to 1 frame per second (fps) for 30 s.

After 30 s the tracking of the larvae was stopped to avoid that larvae reach the outer rim of the plate. The data were recorded using the Multi-Worm Tracker (MWT) software v1.2.0 (Swierczek et al., 2011). The trajectories for every plate were manually tracked using Fiji MTrackJ plug-in (http://fiji.sc). The total distance travelled per 30 s was subsequently analyzed per individual larva using the same software. For each condition 10 larvae were recorded.

### Associative olfactory learning

For the quinine associative olfactory learning experiments, Petri dishes filled with either agarose solution or agarose and 6 mM quinine solution were used. Different agarose concentrations ranging from 0.5 to 3.5% (w/ml) were used in different experiments. As olfactory stimuli, 10 μl amyl acetate (AM, Fluka cat. no.: 46022; diluted 1:50 in paraffin oil, Fluka cat. no.: 76235) and 3-octanol (OCT, undiluted; Fluka cat. no.: 74850) were used. The odorants were loaded into custom-made Teflon containers (4.5-mm diameter) with perforated lids as described in Gerber and Stocker (2007). During training a first group of about 30 animals were exposed to AM (AM+) while crawling on an agarose medium containing 6 mM quinine as a negative reinforcer. After 5 min, larvae were transferred to a fresh Petri dish in which they were allowed to crawl on pure agarose medium for 5 min this time being simultaneously exposed to OCT (OCT). A second group of larvae received the reciprocal training (OCT+, AM). After three training cycles, larvae were transferred onto test plates on which AM and OCT were presented on opposite sides. After 3 min, individuals were counted on the AM side (#AM), the OCT side (#OCT), and the neutral zone on plates containing agarose and quinine solutions. A preference index for each training group was calculated by subtracting the number of larvae on the OCT side from the number of larvae on the AM side and dividing by the total number of counted individuals.

$$\begin{array}{l} {\text{Pref}}_{\text{AM+/OCT}}=(\#\text{AM}-\#\text{OCT})/\#\text{total}\\ {\text{Pref}}_{\text{OCT+/AM}}=(\#\text{AM}-\#\text{OCT})/\#\text{total} \end{array}$$

A Performance Index was calculated from the Preference Indices of the two reciprocally trained groups as follows:

$$\text{PI}=({\text{Pref}}_{\text{AM+/OCT}}-{\text{Pref}}_{\text{OCT+/AM}})/2$$

Negative PIs represent aversive quinine-induced learning.

For experiments in which different agarose concentrations were used as the only reinforcer, a similar experimental design was applied. In detail, one odor was paired with low agarose concentration [0.5% (w/ml)] and another with high agarose concentration [3.5% (w/ml)]. During the test phase, larvae were allowed to choose between the two odors on plates containing 3.5% (w/ml) agarose.

### Statistics

For all experiments that analyze different behaviors of *Drosophila* larvae the data for all different groups were collected in parallel. To compare across multiple groups Kruskal-Wallis test followed by Wilcoxon rank sum test and Holm-Bonferroni correction was performed. Wilcoxon signed ranked test was used to compare one group against chance level. For the quinine diffusion experiment t-*test* was used for comparisons between two groups after confirming that the data are normally distributed. Statistical analysis was performed with R version 2.14.0 and Windows Excel 2010. The data were presented as box plots. The middle line within the box shows the median, the box boundaries refer to the 25 and 75% quantiles, and the whiskers represent the 10 and 90% quantiles. Small circles indicate outliers. Asterisks shown in the figures indicate significance levels: n.s. for *p* > 0.05, ^*^ for *p* < 0.05, ^**^ for *p* < 0.01 and ^***^ for *p* < 0.001.

## Results

### Higher agarose concentrations decrease quinine avoidance

**Figure 1:**
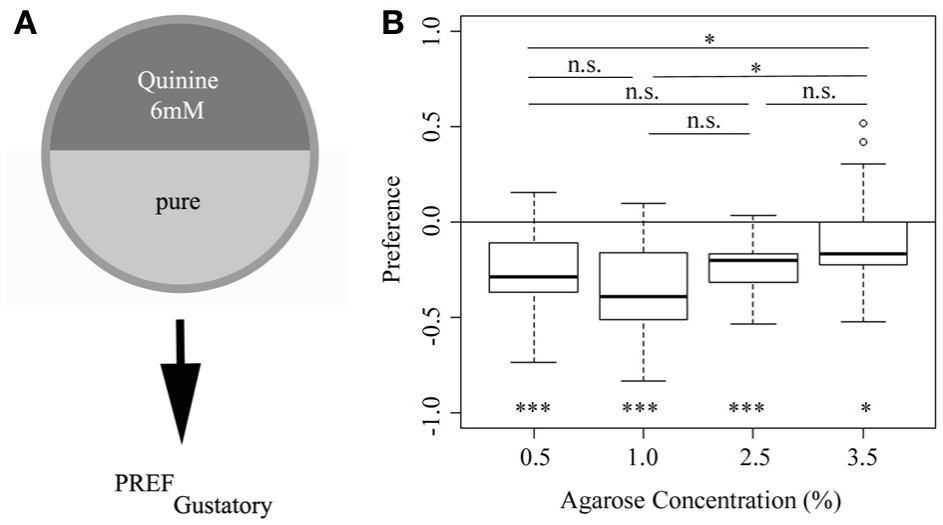
**Larval choice behavior for 6 mM quinine avoidance on different agarose substrates. (A)** Overview of the experimental setup to test for larval choice behavior using 6 mM quinine. **(B)** Wild-type larvae avoid a 6 mM quinine-agarose mixture vs. pure agarose in a choice assay using different agarose concentrations (significance against zero is shown at the bottom of the plot: *p* < 0.001 for 0.5, 1.0, and 2.5% agarose; *p* < 0.5 for 3.5% agarose). Larvae are significantly less repelled, if tested at an agarose concentration of 3.5% compared to 0.5 and 1.0% (*p* < 0.05 in both cases). Thus, the agarose concentration used affects the behavioral output shown in this choice assay. Sample size *n* >12 for each group. Small circles indicate outliers. ^***^p < 0.001; ^*^p < 0.05; n.s., not significant.

The preference (or more accurately behavioral choice) assay used here is a simple paradigm, in which larvae, placed in the center of a Petri dish, are allowed to choose between two different substrates, presented in the two halves of the dish (Aceves-Pina and Quinn, 1979; Rohwedder et al., 2012) (Figure 1A). We have shown in a previous study (Apostolopoulou et al. in preparation) that larvae in this paradigm avoid a 6 mM quinine mixture vs. pure agarose, if an agarose concentration of 2.5% was used. To address whether agarose concentration affects 6 mM quinine avoidance behavior, we applied agarose concentrations ranging from 0.5 to 3.5%. Interestingly, our data show that the agarose concentration can significantly affect quinine avoidance. In particular, a high agarose concentration of 3.5% elicits a significantly lower quinine avoidance response than a concentration of 0.5 and 1.0% (*p* < 0.05 in both cases) (Figure 1B). Thus, in the standard assay used, quinine-dependent avoidance depends on the agarose concentration; and more specifically, at higher concentrations the behavioral response is less pronounced compared to lower concentrations.

### Quinine is homogenously dissolved in the agarose substrates used

**Figure 2:**
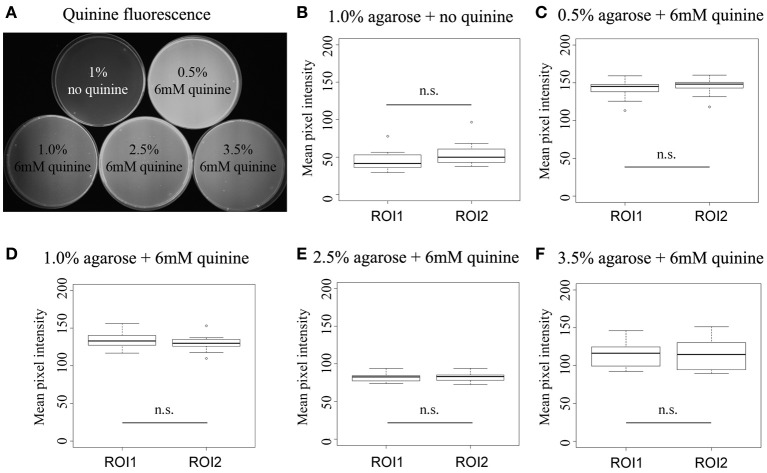
**Quinine is homogenously mixed in the different agarose substrates. (A)** Shows the quinine dependent fluorescence for five different test plates containing only 1% agarose without quinine, 0.5% agarose with 6 mM quinine, 1.0% agarose with 6 mM quinine, 2.5% agarose with 6 mM quinine and 3.5% agarose with 6 mM quinine (from left top to right bottom). **(B–F)** Shows the mean pixel intensity of two defined regions of interest (ROI) of the same size for each type of test plate mentioned above. The mean pixel intensities for the two ROIs on each type of test plate are not significantly different, suggesting a homogenous distribution of quinine within the substrate. Sample size *n* = 10 for each group. n.s., not significant. Small circles indicate outliers.

One concern for these kinds of experiments is of course that quinine—based on its restricted solubility (El-Keredy et al., 2012)—may not be equally distributed within the agarose mixture. So if quinine solubility decreases with increasing agarose concentration one would potentially get a heterogeneous quinine agarose mixture that may change the larval choice behavior similarly as shown in Figure 1B. To control for such an effect we directly traced quinine in the test plates based on its fluorescent emission at about 460 nm (for details see http://www.olympusmicro.com/primer/techniques/fluorescence/fluorescenceintro.html). First, we defined two regions of interest (ROI) of the same pixel size for each test plate and measured the average fluorescence for each ROI per plate (Figure 2A). We mixed 6 mM quinine with 0.5, 1.0, 2.5, or 3.5% agarose and measured 10 plates for each concentration. In addition, we introduced a reference plate of 1% pure agarose without quinine to show that the detected fluorescence depends on the presence of quinine (Figure 2A). The collected data were in each case normally distributed. For each concentration we detected no difference in the mean fluorescence intensity comparing the two ROIs (*p* > 0.05; Figures 2B–F). Thus, we exclude that the less pronounced behavioral response to quinine (Figure 1B) at higher agarose concentrations is due to non-homogenous quinine agarose mixtures.

### Agarose concentration-dependent choice is context-dependent

**Figure 3:**
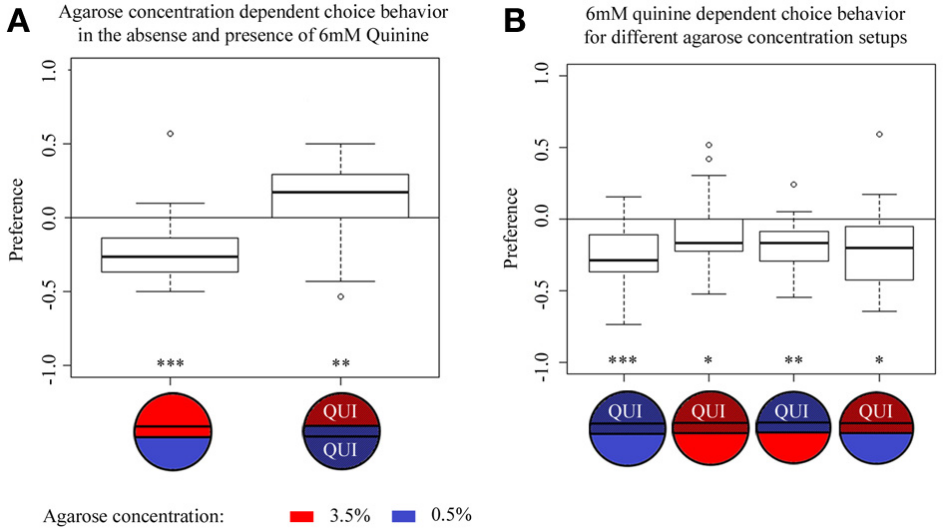
**Agarose- and quinine- dependent choice behavior tested in different contexts. (A)** Agarose-dependent choice behavior for an agarose concentration of 0.5% over 3.5% without quinine (left box plot) or in the presence of 6 mM quinine (right box plot). Without quinine, wild-type larvae avoid 3.5% agarose in favor of the 0.5% agarose side of the Petri dish (*p* < 0.001). In the presence of 6 mM quinine in the entire dish, the preference for the two agarose concentrations reversibly changes compared to the initial situation; now, wild-type larvae avoid 0.5% agarose in favor of 3.5% agarose (*p* < 0.01). **(B)** Larval choice behavior for 6 mM quinine over pure agarose tested on different agarose concentration setups. In general, irrespective of the tested combinations of agarose concentration, wild-type larvae significantly avoid 6 mM quinine over pure agarose, i.e., on plates containing 0.5% agarose on both sides (first boxplot, *p* < 0.001), on plates containing 3.5% agarose on both sides (second boxplot, *p* < 0.05), on plates in which quinine was presented in 0.5% agarose vs. pure 3.5% agarose (third box-plot, *p* < 0.01), and on test plates in which quinine was presented in 3.5% agarose vs. pure 0.5% agarose (fourth box-plot, *p* < 0.05). Sample size *n* >12 for each group. Small circles indicate outliers. ^***^p < 0.001; ^**^p < 0.01; ^*^p < 0.05.

Since different agarose concentrations affect quinine-dependent avoidance, we next asked whether larvae can discriminate between different agarose concentrations *per se*. To test this hypothesis, we filled half of the plate with 3.5% agarose and the other half with 0.5% agarose and allowed wild-type larvae to choose between these two different concentrations. In fact, larvae showed a strong preference for 0.5% agarose over 3.5% agarose (*p* < 0.001 comparing against chance levels) (Figure 3A). Subsequently, we assessed wild-type larvae in a similar agarose concentration preference paradigm, but this time 6 mM quinine was added both to 0.5 and 3.5% agarose sides. To our surprise, the addition of quinine inverted the larval choice behavior in favor of the 3.5% agarose side (*p* < 0.01 comparing against chance levels) (Figure 3A). Thus, agarose-dependent choice behavior turned out to be context-dependent under the conditions tested.

### Quinine is a stronger stimulus than the agarose concentration in a choice assay

We next asked which of the two stimuli, agarose concentration or 6 mM quinine is dominant in a choice assay. In other words, is the quinine-dependent choice behavior also agarose concentration-dependent? (Figure 3B). To answer this question, we performed 6 mM quinine-dependent choice experiments using the following agarose stimuli: 0.5% agarose in the entire Petri dish (first box-plot), 3.5% agarose in the entire dish (second box-plot), 0.5% agarose on the quinine side and 3.5% on the pure side of the dish (third box-plot), 3.5% agarose on the quinine side and 0.5% on the pure side (fourth box-plot). The results of this experiment show that wild-type larvae always avoid the quinine side irrespective of the agarose concentration on the same or the opposite side (Figure 3B, the first and second box plots are the same as shown in Figure 1B). Thus, we conclude that quinine is a stronger stimulus than agarose concentration and that quinine-dependent choice behavior is independent of the applied agarose context.

### Feeding on different agarose concentrations

**Figure 4:**
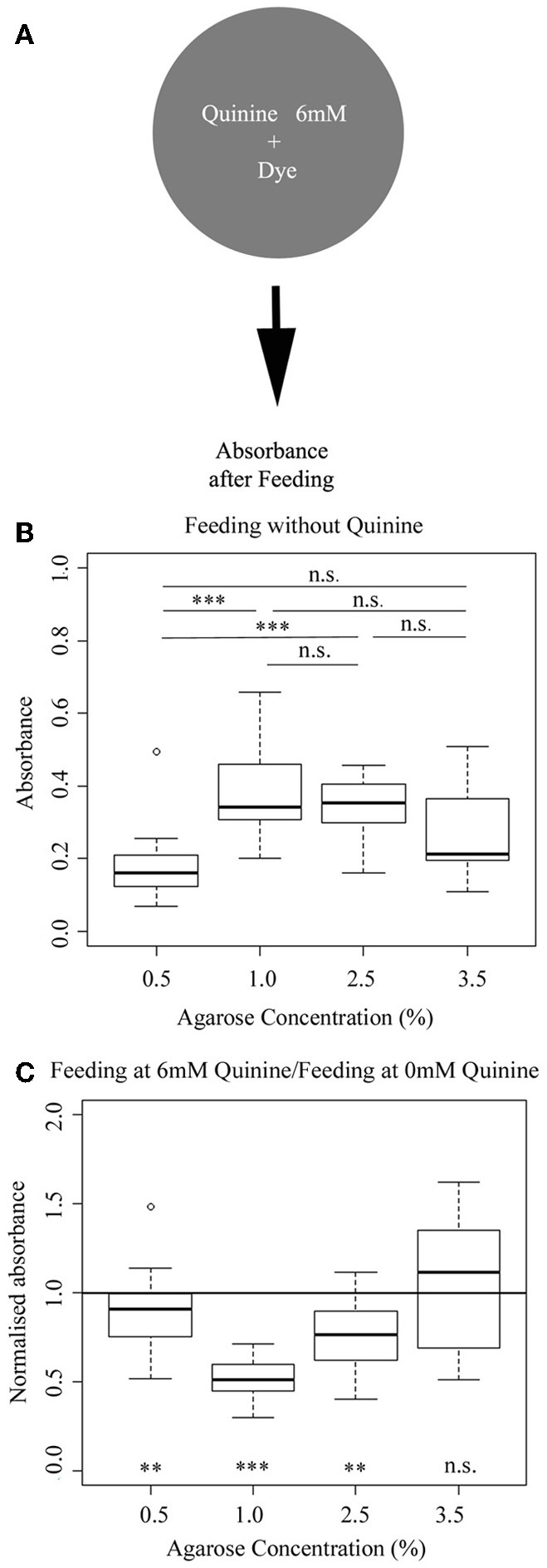
**Larval feeding depends on the concentration of the agarose substrate. (A)** Overview of the experimental setup to test for larval feeding. **(B)** Wild-type larvae show different feeding rates for 0.5, 1.0, 2.5, and 3.5% of agarose. At 0.5% agarose, feeding is lower than feeding on 1.0% agarose (*p* < 0.001) and 2.5% (*p* < 0.001). Feeding at 3.5% agarose is on an intermediate level that is not significantly to any of the other three groups (*p* > 0.05). Differences between individual groups are shown above the box-plots. **(C)** Adding of 6 mM quinine to agarose concentrations tends to reduce feeding compared to the behavior on pure agarose (here visualized by the normalized scores for 6 mM quinine feeding divided by pure agarose feeding). However, the effect is limited to agarose concentrations of 0.5%, 1.0 and 2.5% as only at these concentrations the normalized values are below the baseline feeding observed when the larvae are fed on pure agarose (indicated by 1.0 for normalized absorbance; *p* < 0.01, *p* < 0.001, *p* < 0.01 and *p* > 0.05 for 0.5, 1.0, 2.5, and 3.5%, respectively). A significant difference for each group against normalized absorbance at 1.0 is shown below each box-plot. Sample size *n* >12 for each group. Small circles indicate outliers. ^***^p < 0.001; ^**^p < 0.01; n.s., not significant.

To address a possible effect of different agarose concentrations on feeding behavior, we used a standard assay (Rohwedder et al., 2012). Briefly, we allowed different groups of wild-type larvae to feed on different agarose-concentrated plates, which additionally contained the blue dye indigo carmin. Subsequently, we measured photometrically the absorbance of the larval homogenate as an indirect measurement of the food consumed (Figure 4A). We found that feeding is indeed agarose concentration-dependent. On 0.5% agarose, larvae feed less compared to 1.0 and 2.5% agarose (*p* < 0.001 compared to 1.0% and 2.5%). However, feeding on 3.5% agarose was on a similar low level as on 0.5% agarose (*p* > 0.05 compared to 3.5%), although not significantly different from all other groups (*p* > 0.05; Figure 4B). Thus, our data suggest that larval feeding depends on the agarose substrate used—especially when tested at 0.5% agarose concentration—and there is a trend to show higher levels of food consumption for intermediate concentrations of 1.0 and 2.5%.

### Feeding on different agarose concentrations in the presence of quinine

In the next experiment, we used a similar approach to measure agarose concentration-dependent feeding when 6 mM quinine was added as an aversive substance (Figure 4C). To visualize the quinine-dependent effect on larval feeding for each concentration, we calculated the normalized absorbance for each agarose concentration by dividing the absorbance in 6 mM quinine condition with the absorbance in pure agarose condition (Figure 4C). From our data, we conclude, that the addition of 6 mM quinine strongly reduces feeding, if added in 1% agarose, as in this case feeding is significantly decreased almost by 50% (compared to baseline feeding without quinine indicated by the line at 1.0 normalized absorbance; *p* < 0.001). Within limits, this is also the case if tested at 0.5 and 2.5% agarose (*p* < 0.01 for both). However, addition of 6 mM quinine does not change feeding rate at 3.5% agarose concentration as the normalized feeding is not different from 1.0 (*p* > 0.05). Thus, the quinine-dependent effect on feeding also depends on the agarose concentration used and is basically absent at a substrate concentration of 3.5%.

### Higher agarose concentrations increase larval locomotion

**Figure 5:**
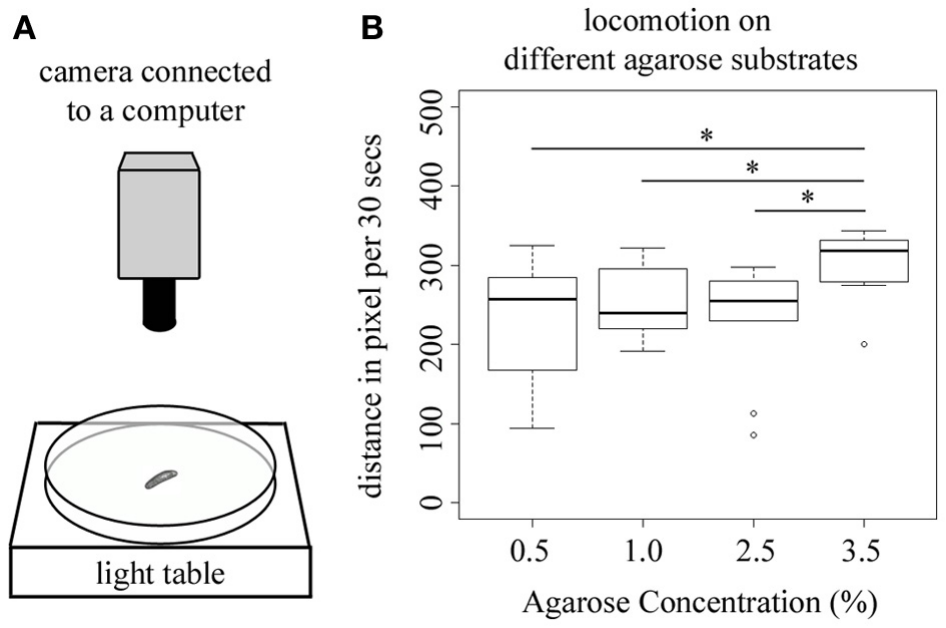
**Larval locomotion depends on the agarose concentration of the substrate. (A)** Overview of the experimental setup to test for larval locomotion. In detail, individual larvae are positioned on a test plate that is located on a light table to allow for a complete illumination of the test plate. The position of the larva is recorded by a video camera that is mounted above the test plate. **(B)** Shows the distance that individual wild-type larva crawl within 30 s on test plates containing 0.5, 1.0, 2.5, or 3.5% agarose. Tracking was restricted to 30 s to exclude that larvae reach the rim of the test plate. At 3.5% agarose concentration larvae significantly increase the distance that they crawl within 30 s (*p* < 0.05 compared to 0.5, 1.0, and 2.5% agarose concentration). Differences between individual groups are shown above the box-plots. Sample size *n* = 10 for each group. Small circles indicate outliers. ^*^p < 0.05.

All larval behaviors tested so far presume that larval locomotion of the animal is not changed by the different agarose substrates during test. Especially, when testing larval choice behavior the position of the animals is only measured at the end of the test, thus an agarose-substrate dependent change in locomotion would most likely directly alter the behavioral output in these experiments. Therefore, we next tested if larvae show differences in locomotion if tested on pure agarose substrates of 0.5, 1.0, 2.5, and 3.5%. In detail, we measured the distance that individual wild-type larva crawl within 30 s. We restricted the tracking to 30 s to exclude that larvae reach the rim of the test plate (Figure 5A). Interestingly, although in our experiments higher agarose concentrations tend to elicit lower behavioral responses, we here observed the opposite effect. At 3.5% agarose concentration larvae showed a significant increase in the distance that they crawl within 30 s (*p* < 0.05 compared to 0.5, 1.0, and 2.5% agarose concentration; Figure 5B). Thus, under the conditions tested larval locomotion also depends on the agarose concentration of the substrate and is significantly increased at higher concentrations.

### Quinine-induced learning takes place only at low agarose concentrations

**Figure 6:**
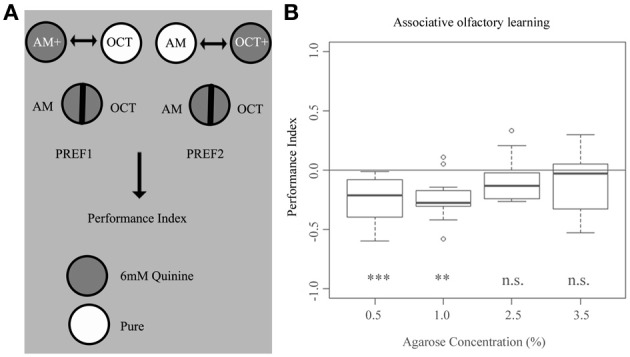
**Larval associative olfactory learning reinforced by 6 mM quinine depends on the concentration of the agarose substrate. (A)** Overview of the experimental setup to test for larval associative olfactory learning reinforced by 6 mM quinine. **(B)** Wild-type larvae show different odor-quinine learning for agarose concentrations of 0.5, 1.0, 2.5, and 3.5%. Only at agarose concentrations of 0.5% and 1.0%, odor-quinine learning leads to the expression of the association (*p* < 0.001 for 0.5% agarose and *p* < 0.01 for 1.0% agarose if tested against zero). No significant learning is detectable when increasing agarose concentration to 2.5 or 3.5% (for both cases *p* > 0.05 if tested against zero). A significant difference for each group against random distribution is shown below each box-plot. Sample size *n* >12 for each group. Small circles indicate outliers. ^***^p < 0.001; ^**^p < 0.01; n.s., not significant.

To investigate if agarose concentration affects 6 mM quinine-reinforced associative olfactory learning (Aceves-Pina and Quinn, 1979; Scherer et al., 2003; Hendel et al., 2005; Gerber and Hendel, 2006; Gerber and Stocker, 2007; El-Keredy et al., 2012), we used a standardized paradigm using agarose concentrations of 0.5, 1.0, 2.5, and 3.5% (Figure 6A). We found that increasing the agarose concentration from 0.5% up to 3.5% significantly changes the output of odor-quinine learning (Figure 6B). For the two lower agarose concentrations (0.5 and 1.0%), odor-quinine learning was significantly different from chance level (*p* < 0.001 and 0.01, respectively), whereas for the two higher concentrations (2.5 and 3.5%) this was not the case (*p* > 0.05 in both cases). Taken together, associative olfactory learning reinforced by 6 mM quinine seems to be context-dependent, as it was only behaviorally expressed at lower agarose concentrations and not expressed at higher agarose concentrations.

### Agarose concentration alone reinforces associative olfactory learning

**Figure 7:**
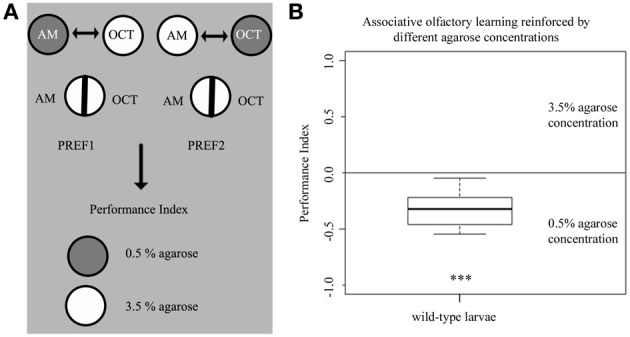
**Different agarose concentrations can be used as reinforcers for larval associative olfactory learning. (A)** Overview of the experimental setup for testing larval associative olfactory learning reinforced by agarose concentrations of 0.5 and 3.5%. **(B)** Wild-type larvae significantly avoid an odor presented on a 3.5% agarose substrate over a second odor presented on a 0.5% agarose substrate (*p* < 0.001). Thus, the agarose concentration of the substrate by itself can serve as a punishing and/or rewarding stimulus. A significant difference for each group against random distribution is shown below each box-plot. Sample size *n* >12. ^***^p < 0.001.

Our data showed so far that larvae can sense the difference between different agarose concentrations and that this information can change various behaviors from choice and feeding to locomotion and learning. Thus, we finally asked if wild-type larvae can associate a given odor with a specific agarose concentration. To answer this question, we modified the learning paradigm used above so that one odor was presented together with 0.5% agarose and a second odor with 3.5% agarose. During the test, larvae were allowed to choose one odor over the other on a 3.5% agarose plate that contained both odors (Figure 7A). In this experiment, larvae showed a preference for the odor associated with the lower 0.5% agarose concentration over the odor that was paired with 3.5% agarose (*p* < 0.001 against chance levels) (Figure 7B). Thus, we conclude that larvae are able to learn differences in the agarose concentration. They prefer an odor that predicts the lower agarose concentration of 0.5% and/or avoid an odor that predicts a higher agarose concentration of 3.5%.

## Discussion

### The effect of the agarose substrate on larval behavior was so far completely neglected

Behavioral experiments in *Drosophila* larvae are usually done by using Petri dishes filled with agar or agarose as a substrate (Gerber and Stocker, 2007). This offers a smooth and soft surface for crawling larvae and at the same time prevents dehydration. Furthermore, the substrate allows the experimenter to add in principle every soluble chemical and test its effect on different larval behaviors like survival, choice behavior, feeding, and learning (Niewalda et al., 2008; Schipanski et al., 2008; El-Keredy et al., 2012; Rohwedder et al., 2012). Due to the transparency and temperature conductivity of the substrate, visually and temperature-guided behaviors can also be analyzed (Gerber et al., 2004; Luo et al., 2010; Keene et al., 2011; Von Essen et al., 2011).

Given the wide use of this experimental substrate, it is surprising that neither the agar/agarose quality nor the concentration is standardized in these experiments. Even more surprising, there are no data available that have analyzed how the substrate itself affects larval behavior. Yet, investigating this issue seems crucial as the heterogeneity of the agar mixture can highly vary depending primarily on the quality and concentration of its agarose and agaropectin components as well as on various other parameters. In this study, we have parametrically analyzed the effect of agarose concentration in the substrate for different larval behaviors. In detail, four gradually increasing agarose concentrations of 0.5, 1.0, 2.5, and 3.5% were used in four well established larval behavioral paradigms: preference, feeding, locomotion and learning.

### Increased agarose concentrations in the substrate reduce the expression of quinine driven behaviors

Larvae avoid quinine, they feed less on a quinine-containing substrate and they can associate an odor with quinine punishment (El-Keredy et al., 2012). However, as we show here, this is only true if tested on a specific agarose substrate. For all three behaviors studied (choice behavior, feeding and associative olfactory learning), larvae show highest responses at a concentration of 1.0% agarose (Figures 1, 4, 6). If tested at a higher concentration of 3.5%, quinine-dependent behavior is either reduced (choice behavior; Figure 1) or not even expressed (feeding, Figure 4; associative olfactory learning, Figure 6). In contrast, we detect the opposite effect for larval locomotion (Figure 5). At a high agarose concentration of 3.5% larvae increase the speed of locomotion compared to lower concentrations. Thus, potentially on rigid substrates larvae try to improve the current situation and due to the lower accessibility of gustatory stimuli increase foraging or reduce the response to aversive gustatory driven stimuli and initiate an escape response. Likely, this effect is further enhanced by another restriction of the substrate as only at lower agarose concentrations—but not at 3.5% agarose concentration—larvae are able to burrow into the substrate (data not shown). Larval burrowing was suggested to be a cooperative behavior that allows larvae to escape predation (Wu et al., 2003; Zhang et al., 2013). In line with this interpretation, increased locomotion was also described in response to different stressful stimuli including low humidity, non-nutritive environments, the texture of the surface of the substrate and noxious heat (Sokolowski et al., 1984; De Belle et al., 1989; Ohyama et al., 2013).

*Drosophila* larvae similar to many limbed organisms largely regulate crawling speed by regulating stride period (Heckscher et al., 2012). Thus, increased locomotion (e.g., foraging and/or escape responses) is based on additional stride cycles within the same time interval that consist of two phases, probes of the substrate with the mouth hooks and muscular contractions passing along the body of the larvae (Sokolowski et al., 1984). So, if larvae increase locomotion each probe with the mouth hooks and its adjacent external and internal sensory organs is shortened and may thus be less sensitive for negative stimuli within the substrate. However, this interpretation is not entirely conclusive as the external sensory organs can in principle collect the same amount of information as they are more often in contact with the substrate. Thus, further experiments are needed to understand how increased locomotion affects quinine driven behaviors based on our initial findings.

### Associative olfactory learning

Interestingly, in contrast to odor-quinine learning, odor-sugar learning was shown to be successful at an agarose concentration of 2.5% for a set of seven different sugars that either offer nutritional benefit or not (Rohwedder et al., 2012). Thus, the agarose dependent effect on learning is different depending on the reinforcer. In line with the conclusion discussed above, it is tempting to speculate that sugar as a positive reinforcer in the substrate may either repress agarose dependent escape responses or changes foraging behavior to enable larvae to establish odor-sugar associations.

In addition, we show that larvae are able to associate an odor with a particular agarose concentration (Figure 7). Unfortunately, our experiments do not reveal if 0.5% agarose is perceived as a reward and/or 3.5% agarose as punishment. With respect to reinforcement processing, it was shown for *Drosophila* larvae and flies that punishment processing is depending on a particular set of dopaminergic neurons (for larvae: likely the DL1 cluster) (Schwaerzel et al., 2003; Riemensperger et al., 2005, 2011; Honjo and Furukubo-Tokunaga, 2009; Mao and Davis, 2009; Selcho et al., 2009; Aso et al., 2010, 2012); whereas appetitive learning was suggested to be processed by the layered organization of octopaminergic and dopaminergic PAM cluster neurons (Schwaerzel et al., 2003; Schroll et al., 2006; Honjo and Furukubo-Tokunaga, 2009; Selcho et al., 2009; Burke et al., 2012; Liu et al., 2012). Thus, specific genetic interference with neuronal function of the larval neuronal circuits that encode reward or punishment will allow to uncover the reinforcing character of the different agarose concentrations. Furthermore, aversive olfactory learning in larvae reinforced by gustatory punishment—but not electric shock (Pauls et al., 2010a)—is only expressed in the presence of the negative reinforcer during test (Gerber and Hendel, 2006; Niewalda et al., 2008; Schleyer et al., 2011; El-Keredy et al., 2012). Hence, olfactory learning reinforced by different agarose concentrations and tested in the presence and absence of negative gustatory reinforcers or at different agarose concentrations will also allow to identify the reinforcing character of the used agarose concentrations.

### Low agarose concentrations in the substrate affect feeding behavior

In addition, not all behavioral effects that are agarose concentration dependent can be described by the expression of escape responses or changes in foraging behavior on rigid substrates. In detail, at 0.5% agarose concentration larval feeding is reduced compared to intermediate concentrations (Figure 4). The data suggests that for larval feeding additional properties of the substrate at low agarose concentrations are also important. So far, several studies suggest that larval feeding is reduced if the substrate is less accessible as it is more solid, contains noxious components or is deleteriously cold (Wu et al., 2005a,b; Lingo et al., 2007). Under the particular conditions that we have tested we would like to expand this list as low agarose concentrations that make the substrate more jellylike show a similar effect. This effect also has to be taken into account when mixing agarose with additional substances that inhibit agarose from polymerizing (e.g., sucralose; data not shown).

### Potential sensory systems involved in sensing and signaling of agarose concentration

As shown by our results, larvae seem to be able to distinguish different concentrations of an agarose substrate and to associate odours with different agarose concentrations (Figures 3, 7). Which sensory systems would allow for such a function? Based on a large number of studies that have analyzed the larval senses, several mechanisms are possible: (i) The larval head region carries three external sensory organs, called dorsal, terminal and ventral organ, which are equipped with gustatory and—for the dorsal organ—with olfactory receptor neurons (Python and Stocker, 2002; Fishilevich et al., 2005; Ramaekers et al., 2005; Colomb et al., 2007; Kwon et al., 2011). In addition, each of these organs also covers a small set of neurons likely involved in mechanosensation (Python and Stocker, 2002). Thus, it is tempting to speculate that these organs may also be able to perceive differences in agarose concentration. (ii) Specialized sensory neurons that tile the larval body wall, the so-called multidendritic neurons, were shown to be involved in sensing and mediating the avoidance response to noxious stimuli (Tracey et al., 2003; Shen et al., 2011). There are four morphologically distinguishable classes of multidendritic neurons, termed class I–IV, based on the complexity of their arborizations (Grueber et al., 2002). Interestingly, class I and II have been suggested to perceive mild mechanical stress, whereas class IV neurons seem to respond to strong thermal and mechanical stress (Hwang et al., 2007; Zhong et al., 2010). (iii) Even more remarkable, recently in class III dendritic arborizations a mechanotransduction channel subunit was identified, called No mechanoreceptor potential C (NOMPC), which mediates gentle touch sensation and seems to be important for environmental exploration (Yan et al., 2013). Thus, based on behavioral description and the established genetic tools to specifically manipulate the neuronal function of each particular sensory system, it is now possible to analyze if these peripheral sensors mediate the perception of agarose concentration.

### Outlook

In this study we show for the first time that different agarose concentrations in the substrate can affect the performance of larval behavioral experiments and in some cases may even be critical for the experimental success. Therefore, direct comparisons between experiments using different agarose concentrations can be misleading. In fact, some of the behavioral phenotypes observed in transgenic animals may rather be related to the substrate specific expression of new behaviors—like escape responses. Thus, a standardization of the parameters in assays measuring larval behavioral preference seems timely.

## Author contributions

Anthi A. Apostolopoulou designed and performed the experiments, analyzed the data and wrote the manuscript. Fabian Hersperger, Annekathrin Widmann, Alexander Wüst, and Lorena Mazija performed the experiments and analyzed the data. Lorena Mazija performed the experiments. Andreas S. Thum designed the experiments, analyzed the data and wrote the manuscript.

### Conflict of interest statement

The authors declare that the research was conducted in the absence of any commercial or financial relationships that could be construed as a potential conflict of interest.
